# Ti surface doping of LiNi_0.5_Mn_1.5_O_4−*δ*_ positive electrodes for lithium ion batteries†

**DOI:** 10.1039/c7ra12932g

**Published:** 2018-02-13

**Authors:** F. Ulu Okudur, J. D'Haen, T. Vranken, D. De Sloovere, M. Verheijen, O. M. Karakulina, A. M. Abakumov, J. Hadermann, M. K. Van Bael, A. Hardy

**Affiliations:** UHasselt, Institute for Materials Research (IMO-IMOMEC), Partner in Energyville, Inorganic and Physical Chemistry Agoralaan, 3590 Diepenbeek Belgium an.hardy@uhasselt.be; UHasselt, Institute for Materials Research (IMO-IMOMEC), Materials Physics Wetenschapspark 1, 3590 Diepenbeek Belgium; EMAT, University of Antwerp Groenenborgerlaan 171 B-2020 Belgium; Skoltech Center for Electrochemical Energy Storage, Skolkovo Institute of Science and Technology Nobel Str. 3 143026 Moscow Russia

## Abstract

The particle surface of LiNi_0.5_Mn_1.5_O_4−*δ*_ (LNMO), a Li-ion battery cathode material, has been modified by Ti cation doping through a hydrolysis–condensation reaction followed by annealing in oxygen. The effect of different annealing temperatures (500–850 °C) on the Ti distribution and electrochemical performance of the surface modified LNMO was investigated. Ti cations diffuse from the preformed amorphous ‘TiO_*x*_’ layer into the LNMO surface during annealing at 500 °C. This results in a 2–4 nm thick Ti-rich spinel surface having lower Mn and Ni content compared to the core of the LNMO particles, which was observed with scanning transmission electron microscopy coupled with compositional EDX mapping. An increase in the annealing temperature promotes the formation of a Ti bulk doped LiNi_(0.5−*w*)_Mn_(1.5+*w*)−*t*_Ti_*t*_O_4_ phase and Ti-rich LiNi_0.5_Mn_1.5−*y*_Ti_*y*_O_4_ segregates above 750 °C. Fourier-transform infrared spectrometry indicates increasing Ni–Mn ordering with annealing temperature, for both bare and surface modified LNMO. Ti surface modified LNMO annealed at 500 °C shows a superior cyclic stability, coulombic efficiency and rate performance compared to bare LNMO annealed at 500 °C when cycled at 3.4–4.9 V *vs.* Li/Li^+^. The improvements are probably due to suppressed Ni and Mn dissolution with Ti surface doping.

## Abstract 2

LiNi_0.5_Mn_1.5_O_4−*δ*_ surface is doped with Ti ion maintaining the spinel structure at 500 °C, higher annealing temperatures cause Ti diffusion from surface towards the core.

## Graphical abstract

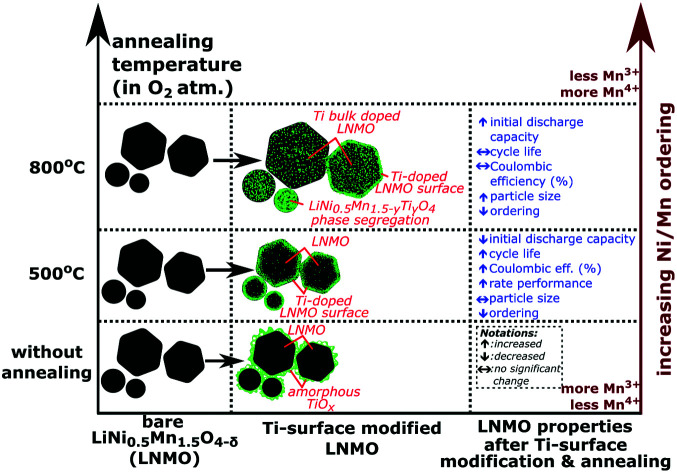

## Introduction

High energy and power density lithium ion batteries (LIB) are extensively studied for their potential applications in portable electronics, hybrid/full electric vehicles as well as for their ability to store solar, wind and other types of renewable energy with high efficiency.^1–4^ Special attention is directed towards electric vehicle research to replace internal combustion engine vehicles to reduce greenhouse gas emissions.^1,5^ Nowadays, LiCoO_2_ is commercially used as a positive electrode (cathode) in LIB for many applications.^2,4^ However, it has a low thermal stability and moreover, cobalt is both toxic and expensive.^1^ Finding a less expensive material, *e.g.* based on manganese, helps reduce the LIB production cost and increase its role in renewable energy applications.^1,6^

LiMn_2_O_4_ (LMO) has a spinel crystal structure containing mixed-valence Mn^4+^ and Mn^3+^, with an average oxidation state of +3.5.^7^ The Mn^3+/4+^ redox reaction is known to take place at the 4.1 V plateau *vs.* Li/Li^+^ during charge/discharge. LMO has the tendency to form oxygen vacancies during high temperature synthesis. Once formed, the oxygen vacancies increase the fraction of Mn^3+^, which, prone to the Jahn–Teller effect, induces lattice expansion and collapse of the structure.^8^ The crystal structure of LMO can be stabilized by substituting Ni^2+^ for Mn^3.5+^, since this increases the average Mn oxidation state. Ni^2+^ substitution results in the LiNi_*x*_Mn_2−*x*_O_4−*δ*_ (LNMO) phase, with a second voltage plateau at 4.7 V corresponding to the Ni^2+/4+^ redox reaction.^9^ This plateau becomes longer as the concentration of Ni^2+^ in LNMO increases, while the Mn^3/4+^ plateau at 4.1 V becomes shorter.^9^ In the stoichiometric LiNi_0.5_Mn_1.5_O_4_ (*x* = 0.5), all Mn remains in its 4+ oxidation state upon cycling and all the energy is stored through Ni redox reactions at a single long 4.7 V plateau. LiNi_0.5_Mn_1.5_O_4_ therefore attracts attention as a high voltage cathode material (4.7 V *vs.* Li/Li^+^) with a good capacity (147 mA h g^−1^) having potential for high power applications.^10,11^

LiNi_0.5_Mn_1.5_O_4_ has two crystalline forms with different degree of ordering of Ni and Mn.^12,13^ In the disordered *Fd*3̄*m* type LNMO Ni and Mn randomly occupy the 16d site.^14,15^ On the other hand, in the fully ordered *P*4_3_32 type LNMO, Ni and Mn reside separately in the 4b and 12d sites, respectively. Formation of the two different crystalline forms is mainly caused by the oxygen evolution reaction and oxygen vacancy formation during anneal. This causes formation of the larger Mn^3+^ cations in comparison to the Mn^4+^, which lead to formation of the disordered LNMO. The structure is influenced by annealing temperature, atmosphere and heating/cooling rates.^12,16^ A mixture of the two can be present as well, defined as LNMO with partial ordering.^17^

The cyclic stability of LNMO still remains an issue since all cathode materials containing Mn are challenged with a capacity fade problem due to Mn leaching into commercial electrolytes, during cycling or storage.^1,18^ The Mn leaching is explained by two different mechanisms: the disproportionation reaction at lower potentials and the acid dissolution at medium to high charged states.^18^ The Mn^3+^ presence in non-stoichiometric LiNi_0.5_Mn_1.5_O_4−*δ*_ may cause Mn^2+^ formation through the following disproportionation reaction:

1 2Mn_solid_^3+^ → Mn_solid_^4+^ + Mn_solution_^2+^

Mn^2+^ dissolves in the electrolyte, while Mn^4+^ remains in the solid. Continuous Mn^2+^ dissolution at the cathode, in combination with a deposition of this Mn^2+^ as metallic Mn at the anode, destabilizes the anode solid electrolyte interface (SEI).^19^ This causes increase of the SEI thickness, irreversible Li^+^ loss and capacity fade in Li-ion full cells, especially with carbon based anodes.^1,18^ The second Mn loss mechanism is by hydrofluoric acid corrosion. HF forms by hydrolysis of LiPF_6_ salt in electrolyte in presence of protonic impurities or traces of water.^18^ LNMO cathode systems are also challenged with a major electrolyte stability problem at high voltages.^20^ A surface layer forms on the cathode–electrolyte interface (CEI), due to electrolyte oxidation reactions at high potentials.^21^ The thickness of this non-conducting^19,20^ layer increases during cycling, causing an increase in impedance and capacity fade, especially at higher temperatures.^20,22,23^

Introducing a protective shell on LNMO that is stable at high voltages can prevent Mn dissolution and increase cycle life. It will also enable good conductivity if the shell is ionically and electronically conductive.^18,22,24–27^ Hao *et al.*^22^ reported Li_4_Ti_5_O_12_ (LTO) and TiO_2_ coatings to effectively reduce the LNMO capacity fade. However, both shell materials provided lower rate performances compared to bare samples, which was explained by low electrical conductivity of TiO_2_ and LTO. As an alternative approach to shell deposition on core materials to suppress Mn dissolution, Lu *et al.* proposed the Ti cation surface doping concept for surface protection of LiMn_2_O_4_ (LMO) particles without altering the spinel surface structure.^28^ They compared Ti surface-doped LMO nanopowders to TiO_2_ surface-coated LMO nanopowders synthesized *via* sol–gel and atomic layer deposition (ALD) techniques, respectively. The surface-doped LiMn_2_O_4_ powders synthesized *via* sol–gel demonstrate a better cyclic stability, electrical and ionic conductivity, compared to TiO_2_ coated LiMn_2_O_4_ synthesized *via* ALD.^28^ A similar Ti surface doping approach was recently reported by Wang *et al.* for LNMO *via* solid-state synthesis using TiO_2_ under a flow of air.^23^ Ti doping took place within a 1–3 nm thick LNMO surface, forming a rocksalt-like structure on the LNMO surface. The rocksalt-like structure was discussed to be a possible blockage for the transfer of Li-ions within the first cycle. Ti surface modification showed no significant improvement on rate performance, cycle life or coulombic efficiency at 25 °C. However, much better capacity retention and coulombic efficiencies were obtained with 55 °C cycling.^23^

In this work, we use a hydrolysis–condensation approach to homogeneously modify the LNMO surface with Ti cation, maintaining the spinel surface structure. Since excessive Ti doping at the core may cause capacity loss,^29^ we investigate the effect of different annealing temperatures (500–850 °C) on Ti diffusion from the surface of LNMO towards the core. Furthermore, we examine the combined effects of annealing temperature, spatial Ti distribution, (dis)order of Ni–Mn ions and particle size on the electrochemical performance.

## Experimental

### Material synthesis

Commercial LiNi_0.5_Mn_1.5_O_4−*δ*_ (LNMO) nanoparticles (Sigma-Aldrich, <500 nm, >99%), also referred to as the ‘bare LNMO’, were used as core material. Bare LNMO powder was annealed at temperatures ranging from 500 to 850 °C for better comparison with the surface modified LNMO, also annealed at temperatures ranging from 500 to 850 °C. The surface modification method reported here is based on TiO_2_ coating made for calcite, α-Fe_2_O_3_, Fe_3_O_4_, SiO_2_, graphene oxide and carbon as core materials.^30,31^ Hydrolysis and condensation reactions of titanium butoxide (TBOT, Aldrich, reagent grade 97%) were catalyzed by NH_3_ (Merck, EMSURE, 25 wt% ammonia solution). 3 g of LNMO as received, was dispersed in 20 mL absolute ethanol (Merck, EMSURE ACS, ISO, Reag. Ph. Eur for analysis) at 25 °C and sonicated for ∼15 minutes in a bath sonicator (Branson, 3510). The dispersion was then added into 80 mL absolute ethanol at 45 °C, under reflux setup, and stirred using a magnetic stirrer bar at 500 rpm. 0.5 mL NH_3_ was added into the suspension. 12 mL TBOT was mixed with 8 mL absolute ethanol. The TBOT–ethanol mixture was added dropwise into the prepared LNMO–NH_3_ ethanol dispersion using an autotitrator (Schott Gerate, T100, TA20) at a rate of 1.43 mL h.^30^ Reactions took place at 45 °C, under a closed N_2_ setup (Air Liquide, Alphagaz 1). The total reaction time was ∼20 hours. After synthesis, the powder was collected through centrifuging (Eppendorf, 5804R) at 14 000*g* for 5 min and washed-centrifuged 3 times with absolute ethanol for 30 min each. Powders were then left to dry overnight at room temperature and ambient atmosphere. Anneals were performed for both bare and surface modified samples at temperatures ranging from 500 to 850 °C, in a tube furnace under O_2_ flow (Air Liquide, Alphagaz Ind.) for only 2 hours to avoid excessive Ti diffusion from surface to core. Heating and cooling rates were 1° min^−1^, corresponding to a total annealing time of 20 to 30 h including the heating and cooling intervals, for annealing temperatures ranging from 500 to 850 °C. Surface modified LNMO powders from the same synthesis experiment were used for all anneals.

### Material characterization

Zeta potential measurements (Brookhaven Instruments, ZetaPALS/90Plus) were carried out for bare LNMO–ethanol dispersions. 5 mL of the sample was taken from a sonicated suspension of 3 g LNMO in 100 mL ethanol, both before and after 0.5 mL NH_3_ addition. The samples were centrifuged at 4000*g* for 2 minutes and the supernatants were collected for zeta-potential measurements. The Hückel approximation was used and the *f*(*K*_a_) value was taken as 1.0 due to the low dielectric constant of ethanol media.^32^ pH measurements in the ethanol media were made according to ASTM standard on pHe determination using a HI 3223 pH meter and water-based HI 1131 electrode.^33^ Thermogravimetric analysis was carried out by means of a Q600 TA Instrument TGA coupled with differential scanning calorimetry (TGA-DSC) from 22 to 800 °C with a heating rate of 10° min^−1^ under dry air flow. The LNMO particle size distribution and morphology were determined using scanning electron microscopy (SEM, FEI Quanta 200F) and transmission electron microscopy (TEM, FEI Tecnai G2 Spirit Twin, 120 kV). ImageJ software was used for data analysis.^34^ TEM samples were prepared by dispersing powder in absolute ethanol, sonicating for ∼30 seconds, dropping onto carbon coated copper grids (EMS, FCF-200-Cu) and drying under an infrared lamp for several minutes. The high angle annular dark field (HAADF) scanning TEM (STEM) imaging was performed at a FEI Osiris (200 kV) and FEI Titan G^3^ microscopes (200 and 300 kV). A Super-X detector was used for the energy dispersive X-ray mapping in a STEM mode (STEM-EDX). The powder X-ray diffraction (XRD) patterns were recorded with a Bruker AXS D8 Discover diffractometer (Cu Kα radiation (*λ* = 1.5418 Å), 0.02° 2*θ* step, LynxEye detector). The lattice parameter refinements were carried out using the GSAS-EXPGUI software.^35,36^ The calibration of the diffractometer constants were done using LaB_6_ powder (Alfa Aesar, 99.5%). Fourier transformed infrared spectroscopy (FTIR) was performed on a Bruker Vertex 70 spectrometer from 4000 to 400 cm^−1^, with 32 scans and 4 cm^−1^ resolution. Pellets containing trace amounts of the sample and 300 mg KBr were prepared by milling in a mortar and pressing under 3 tons for 1.5 minutes. Brunauer–Emmett–Teller (BET) measurements (QuadraSorb SI MP) were made based on N_2_ adsorption at 77 K to determine the surface areas. Samples were degassed at 300 °C for 16 h before measurements.

Coin cells containing Li metal (Sigma-Aldrich), Celgard 2400 separator, working electrode and 1.0 M LiPF_6_ EC/DMC (1 : 1, v/v) electrolyte (Soulbrain) were constructed in an argon glovebox. Working electrodes were prepared by ball-milling of 80 wt% LNMO or surface modified LNMO active material, 10 wt% carbon black (Timcal-imerys c-nergy super C-65) and 10 wt% PVDF (Alfa Aesar, 44080) dissolved in NMP (Alfa Aesar, ACS grade, 99.0+%). The mixture was spread on an aluminum current collector foil by Doctor Blade method (MTI, MSK-AFA-II) after which the foil was dried in a vacuum furnace. Electrode punches of about 3 to 4 mg cm^−2^ loading were made and 1 ton pressure was applied on each for about a second (15 to 20 ìm thickness after drying and aluminum current collector foil thickness subtraction from the total electrode punch thickness). Coin cells were rested for 5 days before measurements. Galvanostatic charge–discharge measurements were performed in 3.4 to 4.9 V voltage window at 0.5 C rate for 200 cycles using a Bio-Logic BCS-805 battery tester. Rate performance measurements were performed within the 0.05 to 2 C rate interval.

## Results and discussion

### LNMO surface modification mechanism

Ti(OBu)_4_ (TBOT) is used as the Ti^4+^ source during synthesis. TBOT is soluble in ethanol and many other organic solvents, but reacts with even traces of water through fast hydrolysis and condensation reactions leading to homogeneous nucleation of solid amorphous TiO_*x*_,^31^ whereas for LNMO surface modification only heterogeneous nucleation is desirable. So, low hydrolysis rates are aimed at in order to obtain uniform amorphous TiO_*x*_ surface modifications and to prevent secondary homogeneous nucleation of amorphous TiO_*x*_. Longer alkyl groups in the alkoxide reduce the partial positive charge on the Ti(iv)-centre and hence lower the hydrolysis rate.^37^ However, here, reaction rates were adjusted by adding 0.5 mL of aqueous NH_3_ acting as a catalyst, in order to control amorphous TiO_*x*_ nucleation at the surface of the core LNMO particles^31^ and to modify a thin layer of the LNMO surface.^31^ A slow TBOT addition rate furthermore provides enough time for the hydrolyzed molecular precursor to diffuse to the surface of the LNMO particles.^30^

Zeta-potential measurements were performed in ethanol surface modification medium to observe the effect of the NH_3_ addition. Results of these measurements are shown in Table 1. NH_3_ addition converts the negative LNMO surface charge to a positive one by the adsorption of NH_4_^+^. The zeta potential of titania in ethanol, on the other hand, was reported to be negative between pH* values of about 5 and 11 (pH* being the operational ‘pH’ measured in ethanol using an ordinary glass electrode with a Ag/AgCl reference electrode in saturated LiCl–ethanol electrolyte).^38^ This probably leads to an electrostatic attraction between negatively charged amorphous TiO_*x*_ species and positively charged LNMO core particles. Wang *et al.*^39^ used a similar *in situ* hydrolysis/condensation based technique to coat polystyrene (PS) nanoparticles with titania and suggested a coating mechanism based mainly on electrostatic attraction. According to this theory, positively charged NH_4_^+^ species are formed and adsorbed on the surface of the negatively charged core PS nanoparticles. The negatively charged hydrolysates of TBOT, (C_4_H_9_OH)_3_TiO^−^, formed in the presence of NH_3_ are then attracted to the core nanoparticle surface by electrostatic attraction between these adsorbed NH_4_^+^ species and titania species. Consequently; condensation reactions, catalyzed by the adsorbed NH_4_^+^, take place at the surface. We propose a similar surface modification mechanism taking place here.

**Table 1:** Zeta-potential measurements for LNMO in ethanol media

Sample	pH	Mean zeta-potential (mV)	±Error
Before 0.5 mL NH_3_ (25 wt%) addition	9.3	−10.8	0.6
After 0.5 mL NH_3_ (25 wt%) addition	10.9	+17.8	1.8

### Material properties at different annealing temperatures

#### LNMO particle size and element distribution

**Fig. 1:**
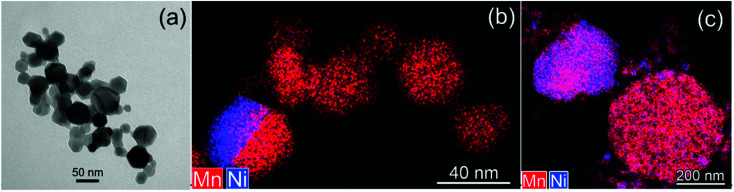
TEM image (a), mixed (Mn, Ni) STEM-EDX maps of the commercial LNMO powder composed of (b) loose 15–50 nm nanoparticles and (c) 200 nm to 2 μm agglomerates.

Commercial LNMO powder was used as core material. The powder consists of loose 15–50 nm nanoparticles and their 200 nm–2 μm agglomerates (Fig. 1). The majority of the 15–50 nm nanoparticles have a composition with a Mn/Ni ratio of 3.0 ± 0.1 as measured by STEM-EDX. However, the Mn/Ni ratio in the agglomerates varies from 1.3 to 3.5, indicating the presence of both Ni-rich and Mn-rich particles (Table S1†). The non-homogeneous elemental distribution can be attributed to the presence of a Ni-rich rock salt impurity phase, Li_*z*_Ni_1−*z*_O, which is also seen by XRD as a minor impurity phase (Fig. S6†). This rock salt impurity phase is commonly present in LNMO powder, also causing Mn enrichment in the main spinel phase.^40^ The chemical composition of the LNMO sample was also analyzed with ICP-AES (Table S2†). Li : Ni : Mn ratios were found to be close to 0.9 : 0.5 : 1.5, indicating an overall agreement with the stoichiometric LiNi_0.5_Mn_1.5_O_4_ formula, while the Li content was slightly lower than the nominal one.

#### TGA-DSC

**Fig. 2:**
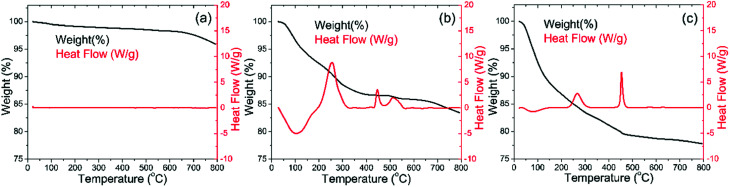
TGA-DSC profiles of the (a) bare LNMO, (b) Ti surface modified LNMO, prior to anneal, synthesized using 12 mL TBOT, 3 mL NH_3_ and 3 g LNMO; (c) amorphous TiO_*x*_ synthesized using 12 mL TBOT and 0.5 mL NH_3_. Exothermic reactions correspond to positive values on the heat flow axis.

TGA-DSC measurements were carried out to determine the temperatures for annealing. Fig. 2 shows the TGA-DSC results for bare LNMO, Ti surface modified LNMO and amorphous TiO_*x*_ powders, all prior to anneal. Surface modified LNMO, synthesized using 3 mL NH_3_ (25 wt%) was used to see the surface modification effect more clearly than when only 0.5 mL NH_3_ (25 wt%) is used. Bare LNMO in Fig. 2(a) does not show any significant changes in heat flow but a weight loss is observed starting from about 700 °C. A similar weight loss at similar temperatures was reported in literature and was attributed to oxygen evolution,^9^ which also results in the formation of the nickel poor LiNi_(0.5−*w*)_Mn_(1.5+*w*)_O_4_ spinel phase. Mn in phase pure LiNi_0.5_Mn_1.5_O_4_ normally has an oxidation state of 4+.^9^ However, within the mentioned nickel deficient spinel phase, Mn^3+^ ions co-exist with Mn^4+^ ions. This can be represented as Li^+^Ni^2+^_*x*_Mn^3+^_1−2*x*_Mn^4+^_1+*x*_O^2−^_4_; where *x* = 0.5 for the phase pure spinel phase and *x* < 0.5 for other cases.^9^

Weight losses due to oxygen evolution can also be observed for the Ti surface modified LNMO, prior to anneal in Fig. 2(b), at temperatures above 700 °C. Two exothermic heat flow peaks at about 250 and 450 °C in Fig. 2(b) are accompanied by weight losses. The DSC curve for amorphous TiO_*x*_ in Fig. 2(c) has similar exothermic heat flow peaks. The two peaks at 250 and 450 °C temperatures are probably an indication of evaporation and combustion of organic compounds or water being released from the unreacted hydroxyl groups.^41^ Based on these results, the minimum annealing temperature was chosen to be 500 °C, in order to remove the organic remains. 850 °C was chosen as the maximum temperature, which is above the oxygen evolution temperature of 700 °C. This allows us to investigate the influence of the oxygen evolution on the crystal structure and battery performance. An oxygen atmosphere was used during all anneals, in order to minimize the initial capacity loss caused by the formation of Li_*z*_Ni_1−*z*_O impurities in oxygen poor ambients.^42^

#### XRD

**Fig. 3:**
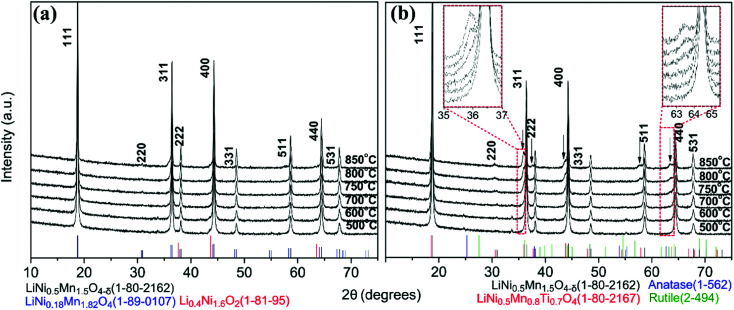
XRD patterns for bare (a) and surface modified (b) LNMO samples synthesized using 3 g LNMO, 12 mL TBOT and 0.5 mL NH_3_, followed by an anneal ranging from 500 to 850 °C. The Bragg positions of different phases are shown with tick marks in different colors.

XRD patterns for bare and surface modified LNMO samples, annealed between 500 and 850 °C, are shown in Fig. 3. Well defined cubic spinel LiNi_0.5_Mn_1.5_O_4−*δ*_ (JCPDS/ICDD 01-80-2162) peaks are observed for bare LNMO samples at all annealing temperatures (Fig. 3(a)). The absence of the LNMO superstructure peak at 15.3° indicates that there is disorder at the (Mn, Ni) site, or at most, only partial order, as further supported by infrared spectroscopy (Fig. 9). Low intensity diffraction peaks are observable for the bare LNMO, above 750 °C (Fig. 3(a)), which could be attributed to the formation of a Mn-rich spinel LiNi_(0.5−*w*)_Mn_(1.5+*w*)_O_4_ phase (*e.g.* LiNi_0.18_Mn_1.82_O_4_ JCPDS/ICDD 01-089-0107) and a Ni-rich rock-salt impurity phase (Li_z_Ni_1−*z*_O, *e.g.* Li_0.4_Ni_1.6_O_2_ JCPDS/ICDD 01-081-95) because of oxygen deficiency.^43^

The XRD patterns of surface modified samples (Fig. 3(b)) show that the LNMO spinel structure was preserved. Secondary phases form in the surface modified LNMO samples at and above 750 °C, which is indicated by the arrows in Fig. 3(b). Following oxygen evolution reaction is proposed for annealing at and above 750 °C, similar to the one proposed by Le *et al.*:^43^

2 LiNi_0.5_Mn_1.5_O_4_ + *a*TiO_*x*(amorp.)_ → *b*LiNi_0.5_Mn_1.5−*y*_Ti_*y*_O_4_ + *c*Li_*z*_Ni_1−*z*_O + *d*LiNi_(0.5−*w*)_Mn_(1.5+*w*)−*t*_Ti_*t*_O_4_ + *e*O_2_

Based on this reaction, the secondary phase peaks in the XRD can be attributed to a combination of three different phases having similar peak positions: the spinel LiNi_0.5_Mn_1.5−*y*_Ti_*y*_O_4_ (*e.g.* LiNi_0.5_Mn_0.8_Ti_0.7_O_4_, JCPDS/ICDD 01-080-2167)^44–46^ and the Mn-rich spinel LiNi_(0.5−*w*)_Mn_(1.5+*w*)−*t*_Ti_*t*_O_4_ (ref. 43) phases formed by replacement of Mn^3/4+^ with Ti^4+^ and the rock-salt Li_*z*_Ni_1−*z*_O impurity phase, formed because of oxygen deficiency. Further discussion on composition of these secondary phases is made in the TEM part.

The lattice parameters of bare and surface modified samples annealed between 500 and 850 °C (Table S3, Fig. S7†^47^) show no significant difference. (HR)STEM was performed for the surface modified samples with 500, 800 and 850 °C anneals to estimate the Ti content and clarify the Ti location in the sample.

#### TEM

**Fig. 4:**
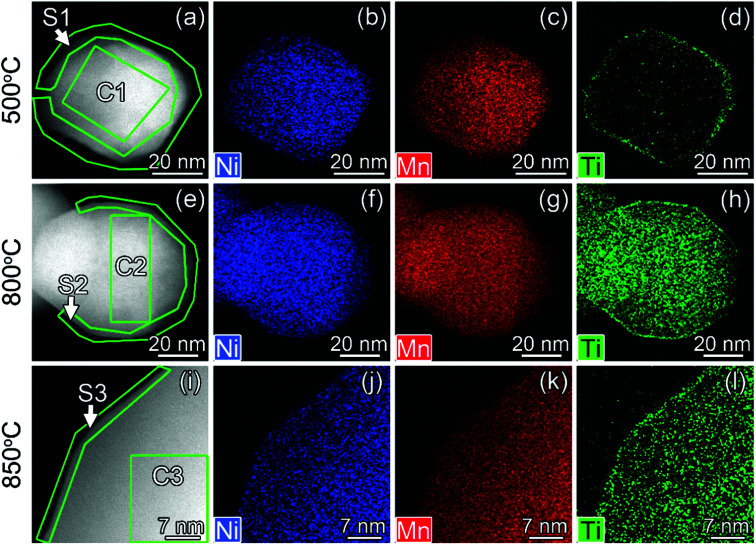
HAADF-STEM images (a, e and i) and STEM-EDX maps of Ni, Mn and Ti for Ti surface-modified LNMO particles annealed at 500 (a–d), 800 (e–h) and 850 °C (i–l). The elemental contents for the marked region are shown in Fig. 5. ‘C’ and ‘S’ stand for ‘core’ and ‘surface’.

**Fig. 5:**
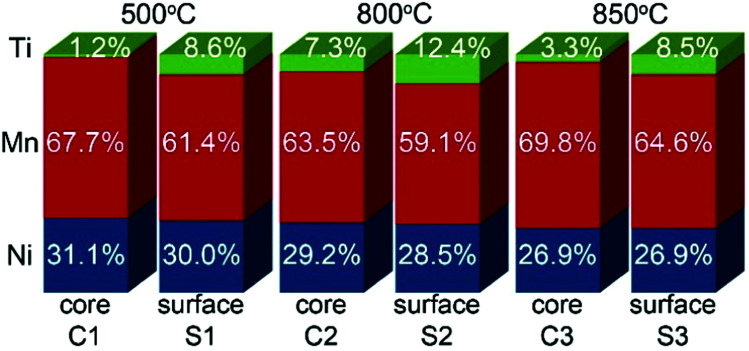
Ni, Mn and Ti contents in atomic% for regions shown in Fig. 4.

Ti surface-modified LNMO particles annealed at 500, 800 and 850 °C were investigated by STEM-EDX (Fig. 4). The surface of the particles differs from the core, having a higher Ti content (Fig. 5). After 500 °C anneal, Ti was predominantly present at the 2–4 nm surface layer (Fig. 4(d)). In the core region, a small amount of Ti was detected, which most probably originates from the Ti-rich surface since TEM provides a 2D projection of a 3D object. An increase in annealing temperature to 800 °C leads to higher Ti diffusion, resulting in 7.3 at% of Ti in the core (Fig. 5).

**Fig. 6:**
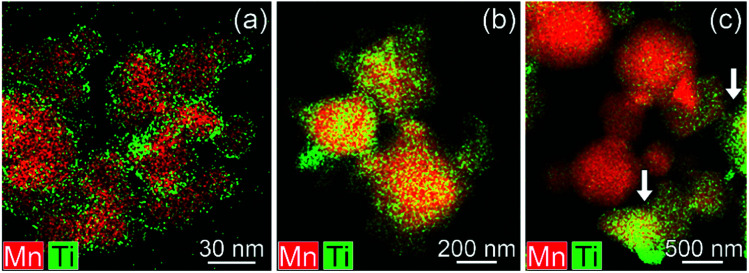
Mixed (Mn, Ti) STEM-EDX maps for Ti surface-modified LNMO annealed at (a) 500, (b) 800 and (c and d) 850 °C. Particles with high Ti content are marked with arrows.

In surface modified LNMO-500 °C, the Ti is mainly present in the surface layer of the LNMO particles (Fig. 6(a)). Only a few Ti-rich impurity particles were noticed. A Ti-containing secondary phase was not observed in the XRD pattern of this sample, probably because the peaks corresponding to the 2–4 nm thick surface layers are too broad and not within the detection limits of powder XRD, or the scattering amount of the Ti modified surface is too small compared to the core of the nanopowders so that possible peaks become hardly detectable. However, Ti-rich particles of about 200 nm and 0.5–1 μm were observed at 800 °C and 850 °C, respectively (Fig. 6(b and c)), which could be attributed to the sharp, secondary phase peaks in their XRDs. The average elemental composition of these Ti-rich particles was estimated for the surface modified LNMO-850 °C (Table 2). Ni content is same as expected for the stoichiometric LiNi_0.5_Mn_1.5_O_4−*δ*_ phase, but the Mn content is lower, resulting in LiNi_0.5_Mn_1.5−*y*_Ti_*y*_O_4_ formula. On the other hand, the majority of the particles contains ∼1 at% of Ti and has lower Ni but higher Mn content compared to the stoichiometric LiNi_0.5_Mn_1.5_O_4−*δ*._ These low Ti content particles can be identified as a Ti bulk doped, LiNi_(0.5−*w*)_Mn_(1.5+*w*)−*t*_Ti_*t*_O_4_ phase.

**Table 2:** Elemental compositions for particles with high or low Ti contents present in surface modified LNMO-850 °C sample

Sample	Ni (at%)	Mn (at%)	Ti (at%)	Mn/Ni
Stoichiometric LiNi_0.5_Mn_1.5_O_4−*δ*_	25	75	—	3

**LNMO particles with:**				
High Ti content	25.0 ± 0.7	69.8 ± 1.8	5.2 ± 1.4	2.8 ± 0.1
Low Ti content	20.8 ± 1.6	78.4 ± 1.9	0.9 ± 0.4	3.8 ± 0.4

**Fig. 7:**
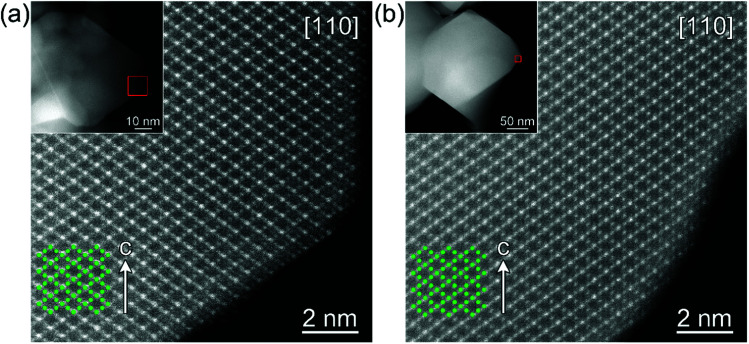
High resolution STEM images of the surface modified LNMO samples annealed at (a) 500, (b) 800 °C. Low magnification images in the insets show the investigated particles and mark the regions presented with atomic resolution.

The high resolution STEM study of the surface modified LNMO samples annealed at 500 and 800 °C shows a well preserved spinel structure (Fig. 7). Rocksalt-like structure formation, reported by Wang *et al.*,^23^ was not observed on the surface; probably due to our choice of O_2_ as the atmosphere during anneals. There was also no separate amorphous or crystalline titania layer at the surface of LNMO after annealing.

**Fig. 8:**
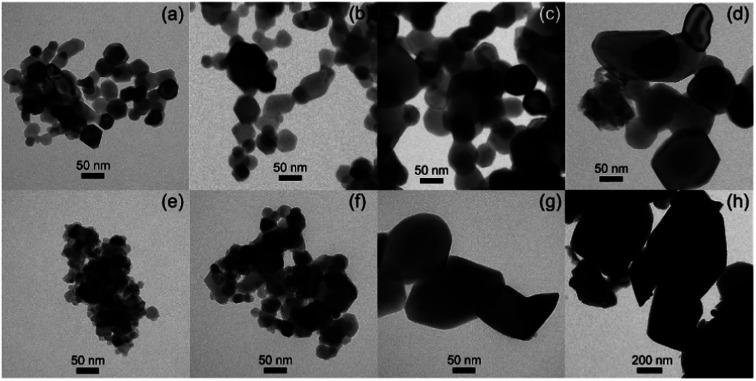
TEM images of bare LNMO after annealing at (a) 500, (b) 700, (c) 800, (d) 850 °C, and of Ti surface modified LNMO annealed at (e) 500, (f) 700, (g) 800, (h) 850 °C.

The particle size of surface-modified LNMO-500 °C remains similar to that for bare LNMO. It can be concluded that the Ti-modified surface layer was kept intact. However, at higher temperatures the particle growth was significant. The particles of surface modified LNMO annealed at 800 °C and 850 °C are ∼1.5–5 times larger than bare LNMO annealed at the same conditions (Fig. 8). It can be suggested that apart from sintering of the particles, an additional process occurs, which is the formation of a Ti bulk doped LNMO phase. Some particles still preserve a 1–2 nm Ti-rich surface layer (Fig. 4(h and l)); while others do not, probably due to their excessive growth during high temperature anneals.

The high Ti content in the surface layer and negligible in the core, the visibility of the spinel structured surface in the HR-STEM images and the absence of a significant lattice parameter increase compared to bare LNMO, all suggest that Ti doping takes place at the 2–4 nm surface without disturbing the spinel structure at 500 °C; rather than formation of a separate, amorphous or crystalline titania layer on the LNMO surface.

#### FTIR

**Fig. 9:**
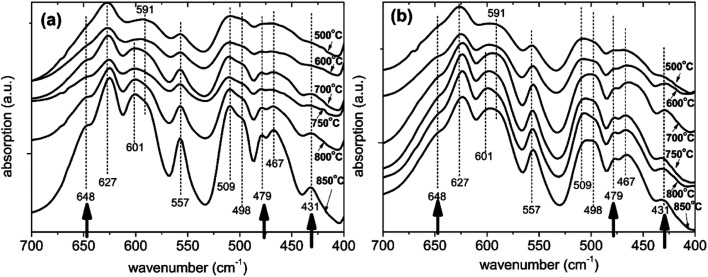
Infrared spectra of (a) bare and (b) surface modified LNMO samples prepared using 3 g LNMO, 6 mL TBOT and 0.5 mL NH_3_ followed by annealing in O_2_ at various temperatures.

Annealing bare LNMO at different temperatures changes cation ordering in the LNMO's crystal structure, probably due to a changing concentration of oxygen vacancies^12^ which also influences the electrochemical performance.^13^ It is difficult to distinguish between the ordered and disordered forms of LNMO by XRD due to close scattering power of Mn and Ni. Vibrational spectroscopy on the other hand is well known to recognize the difference more clearly.^12,48–52^ FTIR measurements were therefore made to probe cation order/disorder. Ordered (P-type) structures have more and better defined FTIR peaks while disordered (F-type) structures have weaker and broader peaks due to disturbances of the long-range order by local lattice distortions.^12^ Kunduraci *et al.*^12^ identified five well-resolved peaks for F-type and eight for P-type LNMO, P-type having three additional peaks compared to the F-type LNMO. FTIR results, similar to those reported by Kunduraci *et al.*^12^ are obtained for bare LNMO, annealed at a range of temperatures, as shown in Fig. 9(a). The bare, 500 °C annealed sample shows five distinctive peaks at 627, 591, 557, 498 and 467 cm^−1^, which are assigned to F-type LNMO as shown in Table 3.

**Table 3:** Peak assignments for the FTIR spectra^a^

Peak number	Disordered LNMO (F-type)		Ordered LNMO (P-type)	
	Peak position (cm^−1^)	Peak assignment^12,48^	Peak position (cm^−1^)	Peak assignment^12,48^
1	627	Mn–O A_1g_	648	—
2	591 (601)	Ni–O F_2g_	627	Mn–O A_1g_
3	557	Mn–O A_1g_	591 (601)	Ni–O F_2g_
4	498 (509)	Ni–O F_2g_	557	Mn–O A_1g_
5	467	—	498 (509)	Ni–O F_2g_
6	—	—	479	—
7	—	—	467	—
8	—	—	431	—

a Shoulder peaks are indicated in parenthesis.

The number of recognizable peaks increases to eight at higher annealing temperatures. The three additional peaks are located at 648, 479 and 431 cm^−1^; as shown with arrows in Fig. 9. This implies more pronounced ordering for bare LNMO by applying higher annealing temperatures in an oxygen atmosphere. The annealing atmosphere has an important effect on ordering as well.^16^ This is further elaborated by Kunduraci *et al.*,^12^ who reported increased disordering at 800 °C when using dry air for the anneal; while increased ordering was obtained when oxygen was used for the study at hand. Our bare LNMO annealed between 600 and 850 °C is identified as a mixture of F and P-types (partial ordering), since pure P-type LNMO (long range ordering) is known to have a more intense 591 cm^−1^ peak compared to the 627 cm^−1^ peak.^12,48^

Fig. 9(b) shows the infrared spectra for the surface modified LNMO samples. The sample annealed at 500 °C is identified as being F-type, similar to the bare LNMO. Spectra for higher annealing temperatures are also similar to the bare ones, showing partial ordering. The 625, 557 and 467 cm^−1^ bands for bare LNMO with 850 °C anneal has shifted to 624, 556 and 465 cm^−1^, respectively, for the surface modified LNMO with 850 °C anneal (Fig. 9). This can be attributed to the presence of small amounts of Ti–O bonds with a similar vibrational wavenumber.^49,53^

### Electrochemical performance

#### Voltage *vs.* capacity curves and d*Q*/d*V* plot

**Fig. 10:**
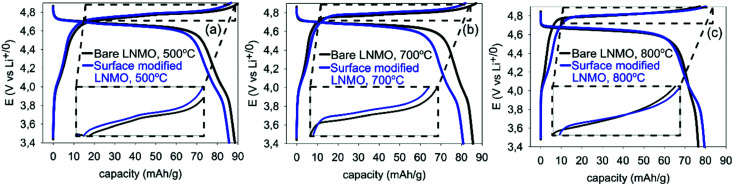
20^th^ cycle galvanostatic charge–discharge curves of half-cells made using bare and surface modified LNMO, annealed at (a) 500, (b) 700 and (c) 800 °C, measured at 0.5 C.

Charge–discharge curves of the 20^th^ cycle of annealed bare LNMO samples and of the corresponding surface modified LNMO samples are shown in Fig. 10 for different annealing temperatures. Low discharge capacities were observed from the 1^st^ until the 19^th^ cycles of bare and surface modified LNMO with 500, 700 or 800 °C anneals, compared to the discharge capacity at their 20^th^ cycles (see Fig. 10). This is probably related to a difficult soaking of the separator with EC/DMC electrolyte.^54^ EC/DMC electrolyte was used since it provided improved coulombic efficiency values compared to when using an EC/DEC electrolyte. The discharge capacity of a bare sample, annealed at 500 °C is about 89 mA h g^−1^ at its 20^th^ cycle, at 0.5 C rate (Fig. 10(a)). Discharge capacities reported for bare LNMO in literature have a close relationship with particle size, size distribution and morphology.^55–59^ Smaller particles are known to undergo more electrolytic side reactions due to higher surface area, causing more capacity loss. Cabana *et al.* (2011) reported initial discharge capacities ranging from 88 to 123 mA h g^−1^ at 1 C for LNMO synthesized using several routes.^55^ Discharge capacities obtained for LNMO powders within this study probably can also be explained by large surface area of the particles and wide particle size distribution.

Surface modified LNMO samples, annealed at 500 or 700 °C, show lower discharge capacities, compared to the bare ones for the first few cycles (see Table 4, Fig. 10). This is consistent with earlier reports on LNMO surface/bulk modifications with Ti cation.^23,26,29,45^ The Li^+^ migration is not hindered by Ti doping as long as the spinel structure is maintained.^29^ However, the electronic migration pathways could be blocked by increasing Ti concentrations for LiNi_0.5_Mn_1.5−*x*_Ti_*x*_O_4_; as proposed by Kim *et al.*^29^ Therefore, increased Ti concentrations in the LNMO might be a possible explanation for the reduced capacities, as the HR-STEM images indicate that the spinel structure is maintained.

**Table 4:** Discharge capacities at the 20^th^ cycles of bare and surface modified samples

Sample	Discharge capacity (mA h g^−1^)			% capacity stored at 4 V region
	Total capacity	4 V region^a^	4.7 V region^b^	
Bare LNMO, 500 °C	88.5	13.6	74.9	15.4
Bare LNMO, 700 °C	85.6	11.3	74.4	13.2
Bare LNMO, 800 °C	76.6	9.0	67.6	11.7
Surface modified LNMO, 500 °C	85.7	14.7	71.0	17.2
Surface modified LNMO, 700 °C	80.9	13.8	67.1	17.1
Surface modified LNMO, 800 °C	79.5	13.3	66.2	16.7

a Includes from 3.4 to 4.4 V.

b Includes from 4.4 to 4.9 V.

Fig. 10 shows that the capacity in LNMO is stored at two different potential regions: one centered around 4.0 V and the other centered around 4.7 V *vs.* Li/Li^+^. Mn^3+^ to Mn^4+^ oxidation takes place during charging at the 4 V region.^60^ However, most of the capacity is stored in the 4.7 V region, where Ni^2+/4+^ oxidation reaction takes place. Table 4 shows the discharge capacities for the 4.0 and 4.7 V regions for bare and surface modified samples. The capacity stored in the 4 V region decreases with annealing temperature for bare samples, due to an increase in ordering (see also Fig. 9). Surface modification of LNMO on the other hand, slightly increases the capacity stored at the 4 V region, especially when annealed at 800 °C (see Table 4). This suggests a slightly higher Mn^3+^ concentration together with more disordering, which is consistent with results reported by Wang *et al.*^23^

**Fig. 11:**
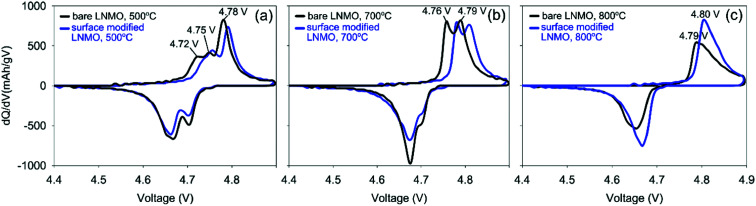
d*Q*/d*V* plots corresponding to the 20^th^ cycle, derived from galvanostatic charge–discharge curves of half-cells made using bare and surface modified LNMO; annealed at (a) 500, (b) 700 and (c) 800 °C.

The two peaks, present in Fig. 11(a) at 4.75 and 4.78 V during charging of bare LNMO, annealed at 500 °C represent the onsets of Ni^2+^ to Ni^3+^ and Ni^3+^ to Ni^4+^ oxidation reactions, respectively.^13,29^ The separation between these two peaks decreases with increasing annealing temperatures, and when annealed at 800 °C the two peaks overlap as one broad peak at a higher potential, as seen in Fig. 11(b) and (c). This is probably an indication of increased cation ordering at higher annealing temperatures.^61^ The increase in redox potential for the ordered variant of LNMO is related to the higher energy required during Li^+^ intercalation/deintercalation.^13,61^Fig. 11(a) shows another peak at about 4.72 V, during charging of the samples annealed at 500 °C. While it is a shoulder peak for the surface modified sample, it is a much more pronounced, well resolved peak in case of bare LNMO. Cho *et al.* observed a similar peak above 4.6 V for LNMO samples with a nanowire morphology.^59^ The peak was attributed to an increased electrolyte decomposition taking place at the cathode surface, due to the high surface area of the nanowires. This corresponds to our LNMO particles, annealed at 500 °C; being significantly smaller than those annealed at higher temperatures such as 700 or 800 °C. The suppression of this peak, which we observe for the surface modified sample in Fig. 11(a), is seen as an indication of a reduced amount of side reactions with Ti modification of the LNMO surface.

#### Cycle life and rate performance

**Fig. 12:**
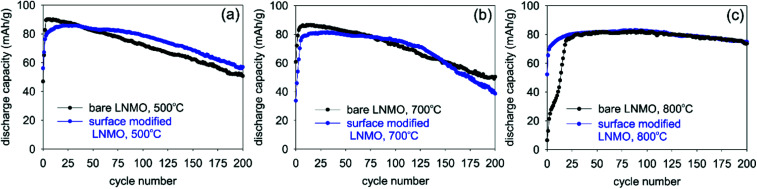
Changes in discharge capacities with increasing cycle numbers for bare and surface modified LNMO samples measured at 0.5 C rate. Samples were annealed at (a) 500, (b) 700 and (c) 800 °C.

Fig. 12 shows the cycle life curves for bare and surface modified LNMO samples annealed at 500, 700 and 800 °C. An early capacity loss is taking place for the bare LNMO samples annealed at 500 and 700 °C. This can be attributed to a significant amount of side reactions taking place at high potentials on the large surface area of these nanoparticles, as well as the dissolution of Mn.^1,20,22^ The surface-modified and 500 °C annealed sample shows a reduced capacity loss. Although the initial discharge capacity is lower, a higher discharge capacity is maintained at the end of 200^th^ cycle compared to the bare LNMO sample (Fig. 12(a)). This improvement can not be linked to a change in particle size after surface modification, since BET measurements suggest similar surface areas of 20 and 24 m^2^ g^−1^ for bare and surface modified LNMO with 500 °C anneals, respectively (Fig. S12†). Reason for this improvement is probably a reduced dissolution of the transition metal (TM) because of the Ti doping. Based on theoretical calculations by Lim *et al.*,^62^ Ti^4+^ ions enhance the TM-oxygen ionic bonding while also increasing the TM dissolution energies. 800 °C bare and surface-modified LNMO samples on the other hand have the best cyclic stabilities due to an increased ordering and low Mn^3+^ concentration (and therefore a limited Mn^3+^ dissolution and resulting capacity fade), as discussed earlier. However, no significant improvement was observed in cycle life with surface modification and 800 °C anneal, compared to its bare counterpart. This might be due to Ti being too much in the bulk rather than on the surface, as well as the large particle size growth.

**Fig. 13:**
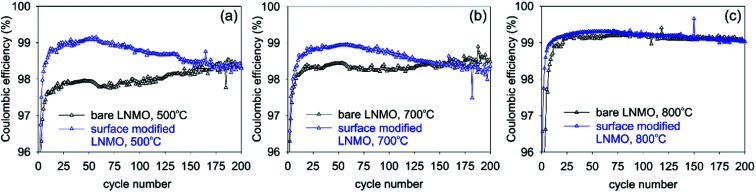
Changes in coulombic efficiencies with increasing cycle numbers for bare and surface modified LNMO samples measured at 0.5 C rate. Samples were annealed at (a) 500, (b) 700 and (c) 800 °C temperatures.

Coulombic efficiency plots (CE% = (*Q*_discharge_/*Q*_charge_) × 100%) are shown in Fig. 13. Low CE% values are observed during the first few cycles of bare LNMO due to SEI and CEI formation on the anode and cathode surfaces, respectively, causing irreversible Li^+^ loss. Efficiencies increase after the formation of these layers. Surface modification improves the CE%, especially for the sample annealed at 500 °C, since this sample has the most disordered structure and the highest amount of Mn^3+^, based on the capacity curves, which results in low CE% and low cycle life, when the surface is not protected. Similar improvements in CE% were reported in literature for surface modified LNMO, especially within the first few cycles. These improvements were attributed to prevention of side reactions and Mn dissolution, causing capacity loss.^22,26^

**Fig. 14:**
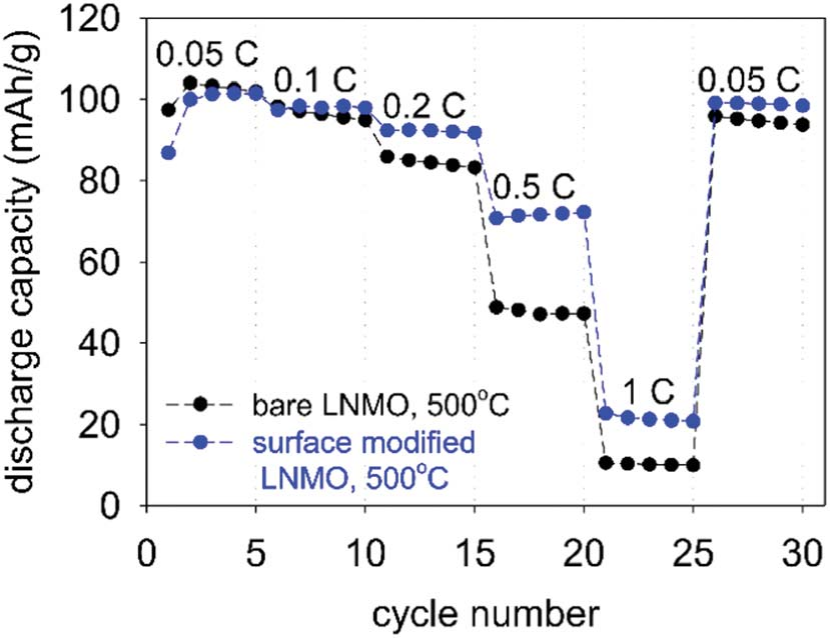
Rate performance measurements for bare and surface modified LNMO, annealed at 500 °C.

Fig. 14 shows rate performance measurements for the bare and surface modified LNMO samples, annealed at 500 °C. The capacity recovery after the final cycle, at 0.1 C, is an indication of preservation of the crystal structure. The surface-modified sample shows a better rate performance than the bare sample. Deng *et al.*^26^ similarly showed an improved rate performance for Li_2_TiO_3_ coated LNMO. However, Hao *et al.*^22^ reported rate performance drops when coating micron sized LNMO with Li_4_Ti_5_O_12_ (LTO). They attributed the lower rate performance to a decrease in electrical conductivity because of the presence of the LTO shell around the particles. Improved rate performances obtained in our case is probably due to preservation of the highly conductive spinel surface structure after Ti surface doping (see Fig. 7), rather than synthesis of a separate titania layer with a different crystal structure.^28^ Small amounts of Ti doping at the surface are useful in preventing crystal structure changes, while also maintaining a high Li^+^ diffusion coefficient during cycling.^28,29,62^

**Fig. 15:**
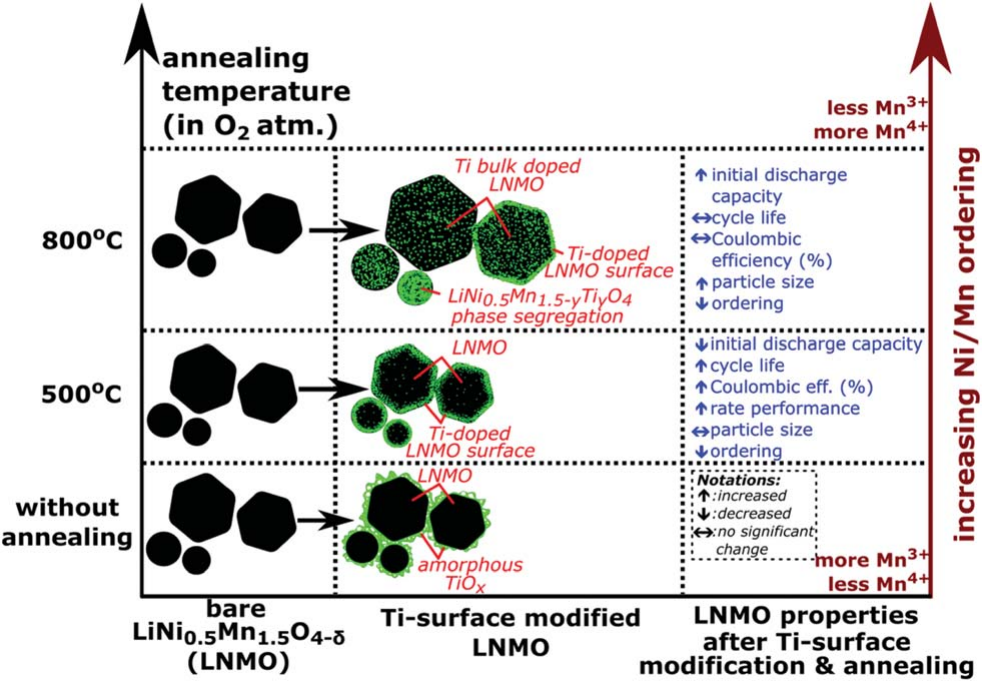
A schematic summary of different annealing temperatures used for Ti surface modified LNMO synthesis and their effect on the Ti positions, particle sizes and the electrochemistry.

Fig. 15 shows a summary of different annealing temperatures used and their effect on the Ti positions, particle sizes and the electrochemistry. A 2–4 nm thick, Ti-rich, spinel solid solution surface forms on LNMO particle by Ti surface doping and 500 °C anneal. Higher annealing temperatures (*i.e.* 800, 850 °C) cause bulk doping of LNMO by Ti (*i.e.* LiNi_(0.5−*w*)_Mn_(1.5+*w*)−*t*_Ti_*t*_O_4_ phase), since the Ti on the surface diffuses towards the core. Three different particle types are observed within the 800 °C annealed sample as shown in Fig. 15: Ti bulk doped LNMO particles that still preserve a 1–2 nm, Ti-rich surface layer; Ti bulk doped LNMO particles without a Ti rich surface layer (or with discontinuities) and Ti-rich segregates (*i.e.* LiNi_0.5_Mn_1.5−*y*_Ti_*y*_O_4_) formed by excessive Ti incorporation into some particles. Cyclic stability, CE% and rate performance is better for the surface modified LNMO than for the bare LNMO with 500 °C anneal, since the Ti-modified surface layer remains intact during annealing. On the other hand, increased Ti diffusion towards the core and particle size growth taking place during 800 °C anneal damages or reduces the thickness of the protective Ti-rich surface layer. Therefore, no significant improvement in cyclic stability or CE% was observed for the surface modified LNMO sample with 800 °C anneal, compared to the bare, 800 °C annealed LNMO.

## Conclusions

By means of a hydrolysis-condensation approach, followed by 500 °C anneal, the surface of a disordered LiNi_0.5_Mn_1.5_O_4−*δ*_ was modified by Ti cation doping over 2–4 nm depth, while maintaining the initial spinel structure. Particle size and surface area of the bare and surface modified LNMO remained similar after 500 °C anneal and the Ti doped surface remained intact. Although the initial discharge capacity was slightly reduced, cycle life, coulombic efficiency and rate performance were improved for Ti surface doped LNMO annealed at 500 °C compared to bare LNMO also annealed at 500 °C. The improvement is probably due to surface structure stabilization by the stronger Ti–O bonds, which reduces the manganese dissolution. On the other hand, during an 800 °C anneal, Ti diffused from the surface towards the core of LNMO, causing the formation of Ti bulk doped LiNi_(0.5−*w*)_Mn_(1.5+*w*)−*t*_Ti_*t*_O_4_ phase and Ti-rich LiNi_0.5_Mn_1.5−*y*_Ti_*y*_O_4_ secondary phase which was accompanied by intensive particle size growth. Mn–Ni ordering in the lattice increased with 800 °C annealing in oxygen for both bare and surface modified LNMO samples, compared to 500 °C annealed samples in oxygen. However, no significant improvement was observed in cycle life or coulombic efficiency of Ti surface modified LNMO annealed at 800 °C compared to bare LNMO also annealed at 800 °C. This is probably because the Ti doped surface layer of LNMO was in this case not well preserved during Ti diffusion and particle size growth. The Ti surface-doped LNMO annealed at 500 °C, having a well preserved spinel surface structure and a disordered Mn–Ni distribution, could be an interesting candidate as a cathode material for lithium ion battery applications requiring both good cycle life and rate performance.

## Conflicts of interest

There are no conflicts of interest to declare.

## Supplementary Material

RA-008-C7RA12932G-s001
