# Supplementary material for: Ti surface doping of LiNi0.5Mn1.5O4−δ positive electrodes for lithium ion batteries
Source: RSC Adv. 2018 Feb 13;8(13):7287–300. doi: 10.1039/c7ra12932g (PMC9078446; doi:10.1039/c7ra12932g)
Supplement: RA-008-C7RA12932G-s001 [file RA-008-C7RA12932G-s001.pdf]

Electronic Supplementary Material (ESI) for RSC Advances.
This journal is © The Royal Society of Chemistry 2018

Supplementary Information

Ti surface doping of $LiNi_{0.5}Mn_{1.5}O_{4-\delta}$ positive electrodes for lithium ion batteries

F. Ulu Okudur [1], J. D'Haen[2], T. Vranken[1], D. De Sloovere[1], M. Verheijen[1], O. M. Karakulina[3], A. M. Abakumov[3,4], J. Hadermann[3], M. K. Van Bael[1], A. Hardy[1]

[1] UHasselt, Institute for Materials Research (IMO-IMOMEC), partner in Energyville, Inorganic and Physical Chemistry, Agoralaan, 3590 Diepenbeek, Belgium
[2] UHasselt, Institute for Materials Research (IMO-IMOMEC), Materials Physics, Wetenschapspark 1, 3590 Diepenbeek, Belgium
[3] EMAT, University of Antwerp, Groenenborgerlaan 171, B-2020 Belgium
[4] Skoltech Center for Electrochemical Energy Storage, Skolkovo Institute of Science and Technology, Nobel str. 3, 143026 Moscow, Russia

Increasing the NH_3 catalyst concentration from 0.5 to 3 mL during synthesis results in increased agglomeration, globular cluster formation within the LNMO particles, with a 500°C anneal (Figure S1). This is an indication of free amorphous TiO_x formation by homogenous nucleation, which crystallizes into TiO_2 during annealing as shown by XRD (Figure S2). The amount of the observable globular clusters reduces and the crystallite shapes change with 700°C annealing of this sample (Figure S1).

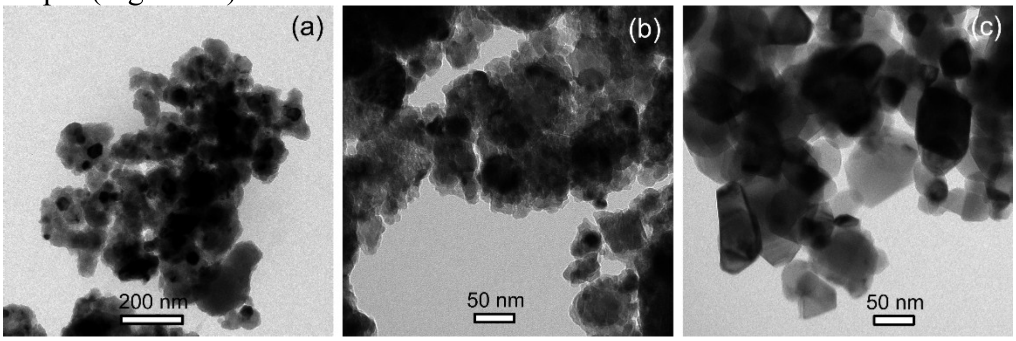

Figure S1. TEM images of surface modified LNMO synthesized using 3 g LNMO, 12 mL TBOT and 3 mL NH_3 (a) without annealing and with annealing at (b) 500°C and (c) 700°C.

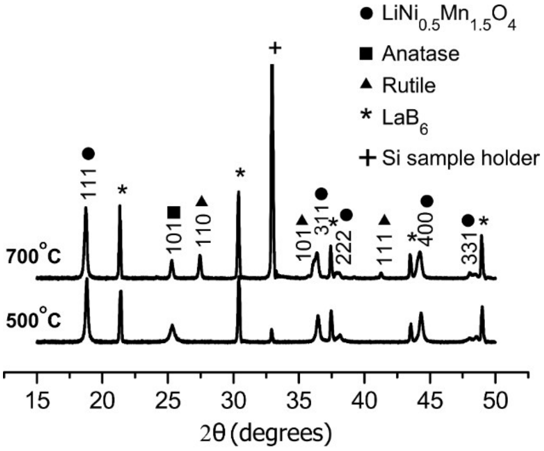

Figure S2. XRD data for surface modified LNMO samples synthesized using 12 mL TBOT and 3 mL NH_3; followed by annealing at 500 and 700°C. High NH_3 (25 wt.%) catalyst concentration caused an increase in the amorphous TiO_x loading; resulting in anatase (JCPDS/ICDD 1-562) formation with 500°C anneal, anatase and rutile (JCPDS/ICDD 2-494) formation with 700°C anneal.

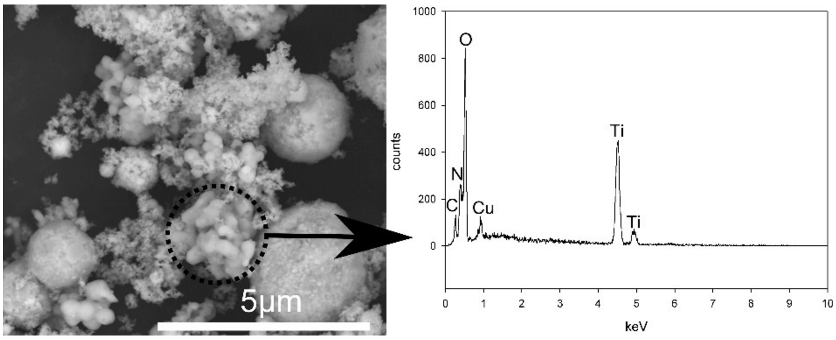

Figure S3. SEM image showing the different morphology of free TiO_2 formed inside LNMO powder during synthesis; using 3 g LNMO, 12 mL TBOT and 3 mL NH_3. Sample was annealed at 700°C temperature (sample dispersion in ethanol, dropping onto 200 mesh copper TEM grid)

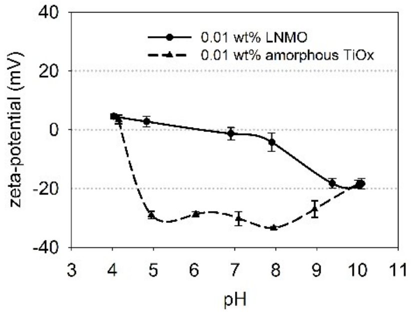

Figure S4. Zeta potential measurements as a fuction of pH, made in 10 mM KNO_3 aqueous suspensions of 0.01 wt.% LNMO and amorphous TiO_x. Each data point was collected as an average of minimum 3 runs. An important observation is that the surface charge at basic pHs is negative, both for LNMO and amorphous TiO_x. Amorphous TiO_x was synthesized using 12 mL TBOT and 0.5 mL NH_3.

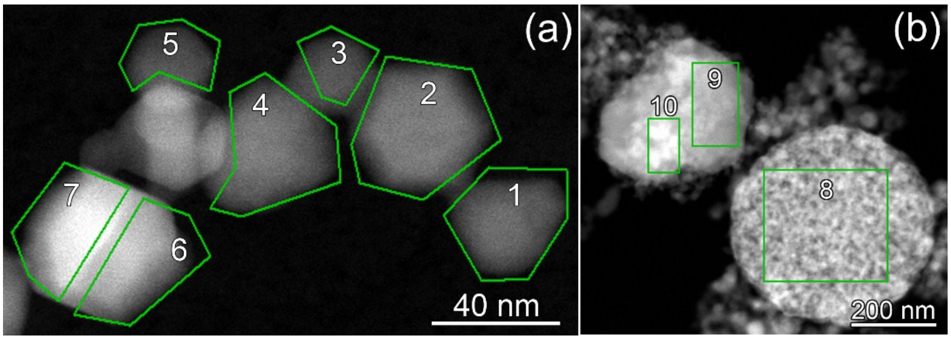

Figure S5. HAADF-STEM images of the commercial LNMO powder composed of (a) loose 15-50 nm nanoparticles and (b) 200 nm - 2 µm agglomerates.

Table S1. The Mn/Ni ratios from commercial LNMO measured by STEM-EDX from regions indicated in Figure S5.

	Mn/Ni	
**Region**	**15-50 nm particles**	**200 nm – 2 μm particles**
1	3.1	-
2	3.0	-
3	2.9	-
4	3.0	-
5	2.8	-
6	2.9	-
7	0.3	-
8	-	3.5
9	-	1.3
10	-	1.8

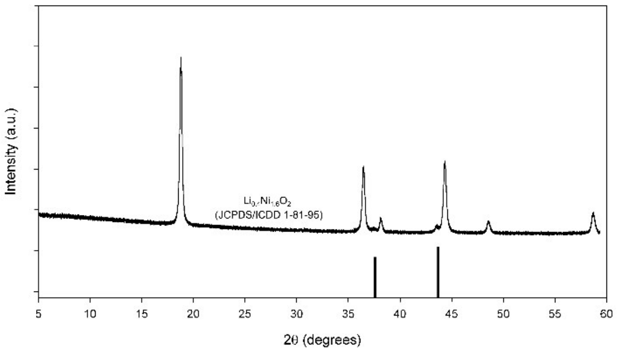

Figure S6. PXRD pattern of the commercial LNMO.

Table S2. Chemical composition analysis results by ICP-AES for bare (commercial) LNMO without any anneals and for bare and surface modified LNMO with anneals at different temperatures

Sample	Measurement	Li (wt%)	Li (mol)	Ni (wt%)	Ni (mol)	Mn (wt%)	Mn (mol)	Ti (wt%)	Ti (mol)	Stoichiometry (based on Ni standardization)	Li:Ni:Mn:Ti (average molar ratio)
bare (comm.) LNMO	1	3.35	4.81E-04	15.4	2.63E-04	44.2	8.05E-04	-	-	$Li_{0.91}Ni_{0.50}Mn_{1.53}O_x$	0.91:0.5:1.51
	2	3.25	4.67E-04	15.2	2.59E-04	42.4	7.72E-04	-	-	$Li_{0.90}Ni_{0.50}Mn_{1.49}O_x$	
bare LNMO-500°C	1	3.50	5.03E-05	17.0	2.89E-05	46.0	8.37E-05	-	-	$Li_{0.87}Ni_{0.50}Mn_{1.45}O_x$	0.87:0.5:1.45
	2	3.53	5.08E-05	17.1	2.91E-05	46.4	8.45E-05	-	-	$Li_{0.87}Ni_{0.50}Mn_{1.45}O_x$	
bare LNMO-800°C	1	3.55	5.10E-05	16.5	2.81E-05	45.2	8.23E-05	-	-	$Li_{0.91}Ni_{0.50}Mn_{1.46}O_x$	0.92:0.5:1.49
	2	3.52	5.06E-05	16.0	2.73E-05	45.8	8.34E-05	-	-	$Li_{0.93}Ni_{0.50}Mn_{1.53}O_x$	
surface mod. LNMO-500°C	1	3.32	4.78E-05	15.2	2.58E-05	44.6	8.11E-05	1.44	3.01E-06	$Li_{0.92}Ni_{0.50}Mn_{1.57}Ti_{0.06}O_x$	0.92:0.5:1.54: 0.06
	2	3.38	4.86E-05	15.4	2.63E-05	43.3	7.89E-05	1.47	3.07E-06	$Li_{0.92}Ni_{0.50}Mn_{1.50}Ti_{0.06}O_x$	
surface mod. LNMO-800°C	1	3.26	9.37E-06	14.9	5.07E-06	42.9	1.56E-05	2.08	8.70E-07	$Li_{0.92}Ni_{0.50}Mn_{1.54}Ti_{0.09}O_x$	0.92:0.5:1.53: 0.08
	2	3.22	9.28E-06	14.9	5.07E-06	42.5	1.55E-05	1.69	7.04E-07	$Li_{0.91}Ni_{0.50}Mn_{1.53}Ti_{0.07}O_x$	

Table S3. Lattice parameter refinement results and crystallite size calculations obtained from XRD data of bare and surface modified LNMO annealed at different temperatures.

Sample	a (Å)	$\%R_{wp}$	$\%R_{Bragg}$	χ^2	L_x	Average crystallite size (nm)
surface mod. LNMO-500°C	8.18170(10)	3.19	1.96	1.449	14.98	53
surface mod. LNMO-600°C	8.18269(21)	3.26	2.17	1.520	15.22	52
surface mod. LNMO-700°C	8.18551(19)	3.51	2.18	1.753	7.36	108
surface mod. LNMO-750°C	8.18594(7)	3.15	1.49	1.433	4.23	188
surface mod. LNMO-800°C	8.18574(7)	3.64	3.57	1.876	3.67	216
surface mod. LNMO-850°C	8.18547(7)	3.65	2.16	1.903	0.82	967
bare LNMO-500°C	8.17909(19)	3.04	1.61	1.331	13.33	60
bare LNMO-600°C	8.17913(19)	3.01	2.00	1.314	16.31	49
bare LNMO-700°C	8.17952(7)	2.87	1.51	1.180	10.24	78
bare LNMO-750°C	8.18100(6)	3.01	2.16	1.275	8.36	95
bare LNMO-800°C	8.18184(6)	3.46	2.20	1.691	3.86	206
bare LNMO-850°C	8.17976(12)	3.87	2.40	2.224	4.16	191

Table S4. Atom sites used for both $LiNi_{0.5}Mn_{1.5}O_{4-\delta}$ and $LiNi_{0.5}Mn_{1.5-y}Ti_yO_4$ lattice parameter refinements [1] (Ti was assumed to replace Mn at the 16d sites [2])

atom	site	x	y	z	site occupancy
Li	8a	0.125	0.125	0.125	1
Ni	16d	0.5	0.5	0.5	0.25
Mn	16d	0.5	0.5	0.5	0.75
O	32e	0.26314(6)	0.26323(5)	0.2632(7)	1

[1] Branford, W., Green, M.A., Neumann, D.A., Structure and ferromagnetism in Mn(4+) spinels: A $M_{0.5}$ $Mn_{1.5}$ O_4 (A = Li, Cu; M = Ni, Mg). Chem. Mater., 2002. ***14****: p. 1649-1656.*

[2] Le, M.-L.-P., Strobel, P., Colin, C., Pagnier, T. and Alloin, F. Spinel-type solid solutions involving Mn4+ and Ti4+:Crystal chemistry, magnetic and electrochemical properties. Journal of Physics and Chemistry of Solids, 2011. ***72****(2): p. 124-135.*

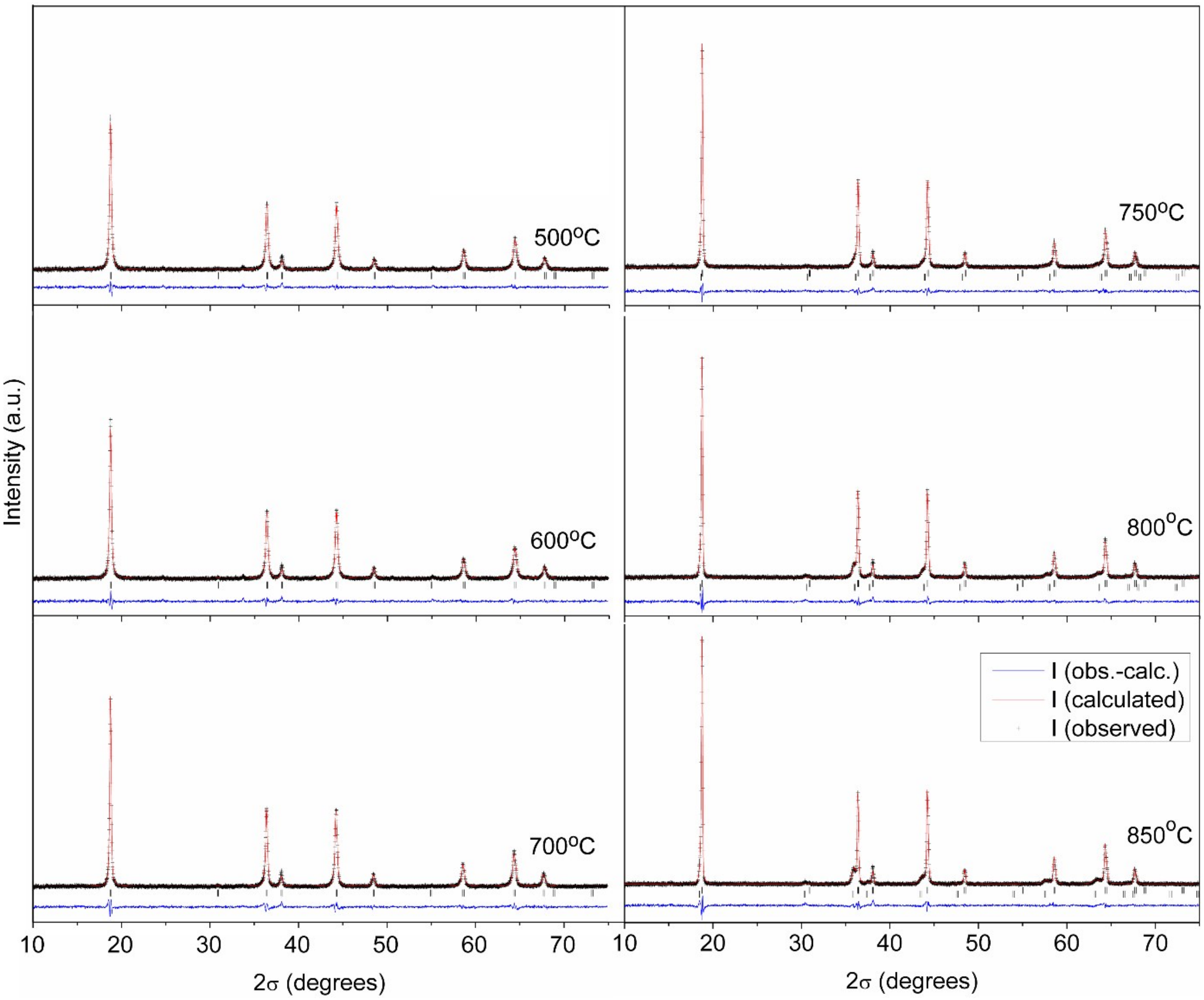

Figure S7. Refinement profiles for surface modified LNMO samples prepared using 3 g LNMO, 12 mL TBOT and 0.5 mL NH3 (25 wt.%), followed by anneals at different temperatures. The Bragg positions of $LiNi_{0.5}Mn_{1.5}O_{4-\delta}$ and $LiNi_{0.5}Mn_{1.5-y}Ti_yO_4$ phases are shown as tick marks located in upper and lower positions, respectively.

Table S5. Particle diameter measurements based on TEM images (30-2700 x magnifications)

Commercial LNMO		Surface mod. LNMO, 500°C		Surface mod. LNMO, 800°C		Surface mod. LNMO, 850°C	
**#**	**diameter (nm)**	**#**	**diameter (nm)**	**#**	**diameter (nm)**	**#**	**diameter (nm)**
1	15.944	**1**	21.809	**1**	62.618	**1**	242.249
2	16.582	**2**	22.989	**2**	67.08	**2**	338.151
3	18.005	**3**	24.866	**3**	70.562	**3**	357.693
4	19.133	**4**	26.809	**4**	72.593	**4**	366.788
5	21.287	**5**	26.907	**5**	74.031	**5**	379.958
6	21.429	**6**	27.682	**6**	78.002	**6**	425.512
7	22.395	**7**	29.078	**7**	78.186	**7**	436.651
8	22.862	**8**	29.885	**8**	84.876	**8**	443.005
9	24.766	**9**	30.67	**9**	88.403	**9**	478.273
10	26.156	**10**	33.154	**10**	90.203	**10**	499.664
11	26.427	**11**	34.559	**11**	91.472	**11**	593.319
12	26.634	**12**	36.348	**12**	96.42	**12**	630.629
13	26.907	**13**	37.068	**13**	97.923	**13**	647.912
14	27.298	**14**	38.26	**14**	100.921	**14**	659.789
15	27.807	**15**	40.148	**15**	106.983	**15**	677.489
16	28.321	**16**	42.264	**16**	109.327	**16**	680.846
17	31.671	**17**	42.389	**17**	111.799	**17**	712.422
18	32.998	**18**	42.732	**18**	113.58	**18**	721.63
19	33.072	**19**	43.618	**19**	118.264	**19**	737.223
20	33.808	**20**	43.919	**20**	118.485	**20**	754.125
21	34.587	**21**	43.919	**21**	119.724	**21**	806.878
22	34.868	**22**	44.159	**22**	121.962	**22**	823.101

23	36.996	**23**	44.636	**23**	125.387	**23**	891.579
24	37.86	**24**	45.977	**24**	126.568	**24**	1024.652
25	39.793	**25**	46.548	**25**	127.267	**25**	1240.571
26	42.458	**26**	46.888	**26**	127.896	**26**	1257.456
27	43.204	**27**	47.168	**27**	130.004	**Avg.**	**647**
28	43.241	**28**	49.752	**28**	130.236		
29	44.024	**29**	49.752	**29**	131.246		
30	44.78	**30**	50.36	**30**	140.021		
31	44.921	**31**	52.523	**31**	144.665		
32	45.568	**32**	53.321	**32**	145.911		
33	46.923	**33**	54.239	**33**	146.049		
34	51.418	**34**	58.609	**34**	150.063		
35	54.841	**35**	58.609	**35**	153.239		
36	66.061	**36**	61.77	**36**	158.114		
37	211	**37**	62.451	**37**	164.699		
38	236	**38**	63.915	**38**	165.92		
39	249	**39**	66.825	**39**	176.652		
40	249	**40**	66.962	**40**	179.811		
41	282	**41**	67.298	**41**	201.74		
42	319	**42**	69.338	**42**	215.377		
43	325	**43**	71.669	**43**	220.379		
44	325	**44**	73.088	**44**	247.5		
45	374	**45**	73.088	**45**	253.973		
46	381	**46**	74.136	**46**	264.447		
47	441	**47**	75.03	**47**	287.529		
48	479	**48**	75.162	**48**	288.444		
49	557	**49**	77.19	**49**	317.128		
50	557	**50**	80.755	**50**	350.903		
51	578	**51**	81.342	**51**	355.258		
52	646	**52**	86.055	**52**	402.87		
53	658	**53**	89.744	**53**	407.922		
54	819	**54**	89.972	**54**	441.603		
55	837	**55**	100.72	**55**	447.145		
56	924	**56**	159.188	**56**	467.718		
57	1047	**57**	187.155	**57**	479.383		
58	1150	**58**	292.984	**58**	496.258		
59	1577	**59**	332.498	**59**	508.652		
**Avg.**	**245**	**60**	411.556	**60**	523.161		
		**61**	436.663	**61**	528.242		
		**62**	461.85	**62**	579.545		
		**63**	464.378	**63**	648.892		
		**64**	495.496	**64**	664.326		
		**65**	547.42	**65**	669.901		
		**66**	738.607	**66**	676.391		
		**67**	760.096	**67**	701.807		
		**68**	790.79	**68**	830.152		
		**69**	899.3	**69**	851.34		
		**70**	1064.97	**70**	876.737		
		**71**	1099.226	**71**	1023.05		
		**72**	1191.807	**72**	1088.128		
		**73**	1542.807	**73**	1371.248		
		**74**	1632.547	**74**	1462.358		
		**75**	1756.457	**75**	1476.835		
		**Avg.**	**243**	**Avg.**	**338**		

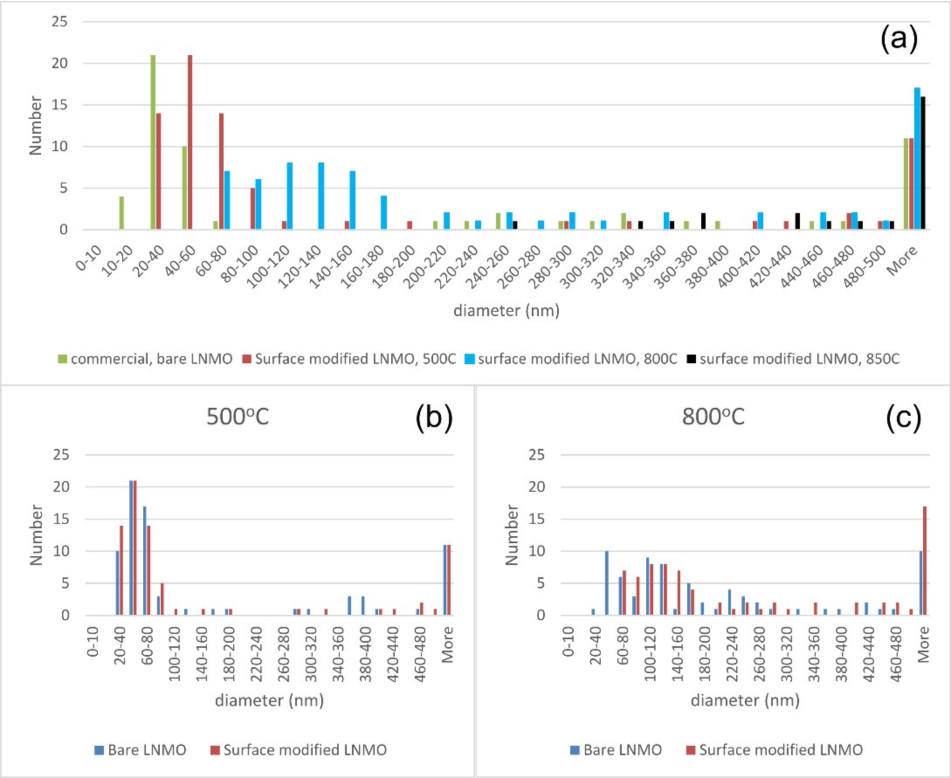

Figure S8. (a) Particle size distribution diagram for bare (commercial)LNMO without any anneals and Ti surface modified LNMO annealed at different temperatures. (b)(c) Particle size distributions for the bare (commercial)LNMO with 500 or 800°C anneals; and Ti-surface modified LNMO samples, with 500 or 800°C anneals. Measurements were made using TEM images
(30-2700 x magnifications)

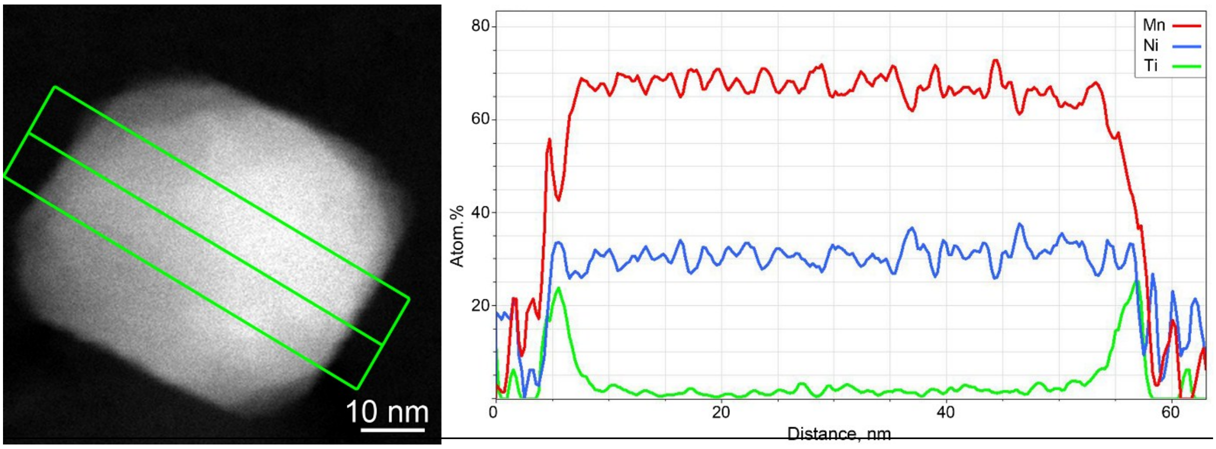

Figure S9. HAADF-STEM image of surface modified LNMO annealed at 500°C (left). The green line marks the region used to plot the profiles of atomic content of Mn, Ni and Ti (right). At the surface layer (2-3 nm) Ti concetration is higher.

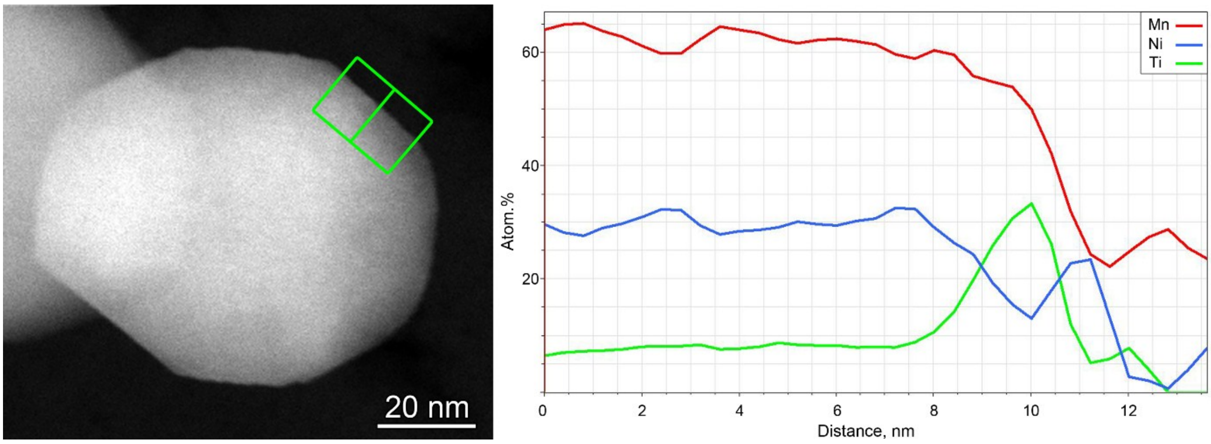

Figure S10. HAADF-STEM image of surface modified LNMO annealed at 800°C (left). The green line marks the region used to plot the profiles of atomic content of Mn, Ni and Ti (right). At the surface layer (2 nm) Ti concetration is higher.

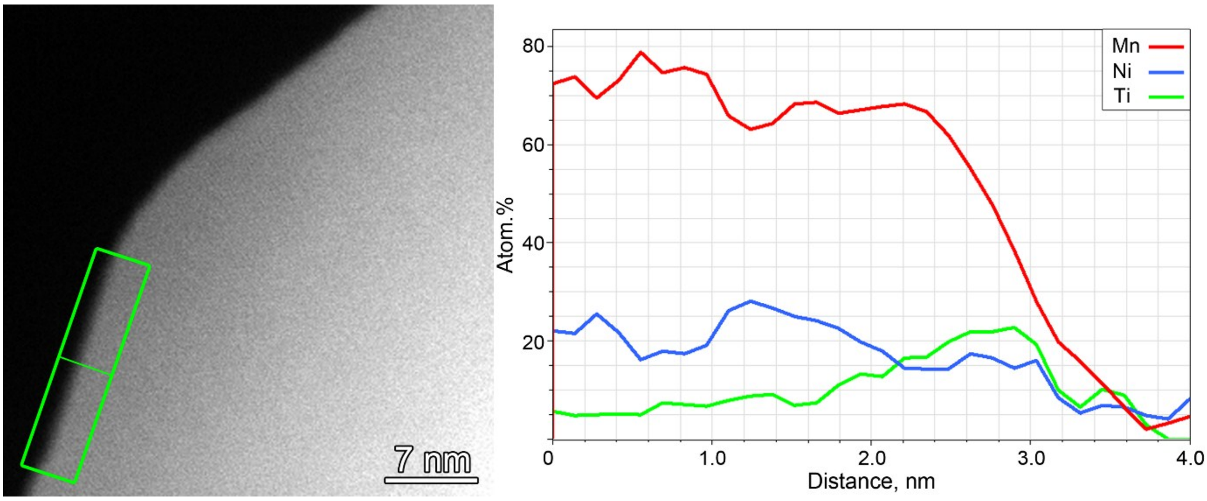

Figure S11. HAADF-STEM image of surface modified LNMO annealed at 850°C (left). The green line marks the region used to plot the profiles of atomic content of Mn, Ni and Ti (right). At the surface layer Ti concetration is higher.

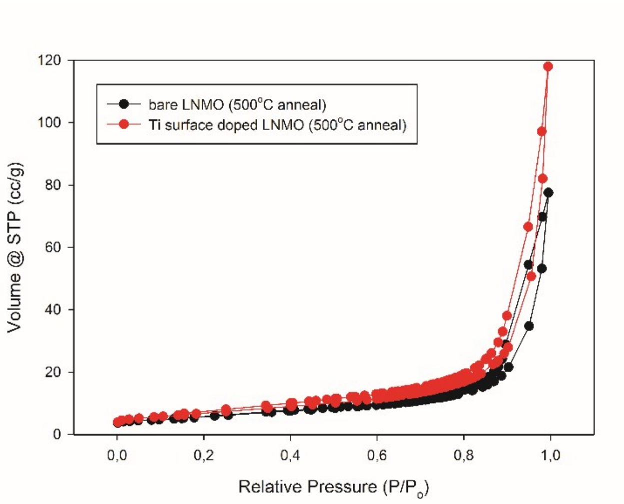

Figure S12. BET measurements for bare and Ti surface modified LNMO with 500°C anneals. Surface areas were determined to be 20 and 24 m^2/g for bare and Ti surface modified LNMO samples, respectively.

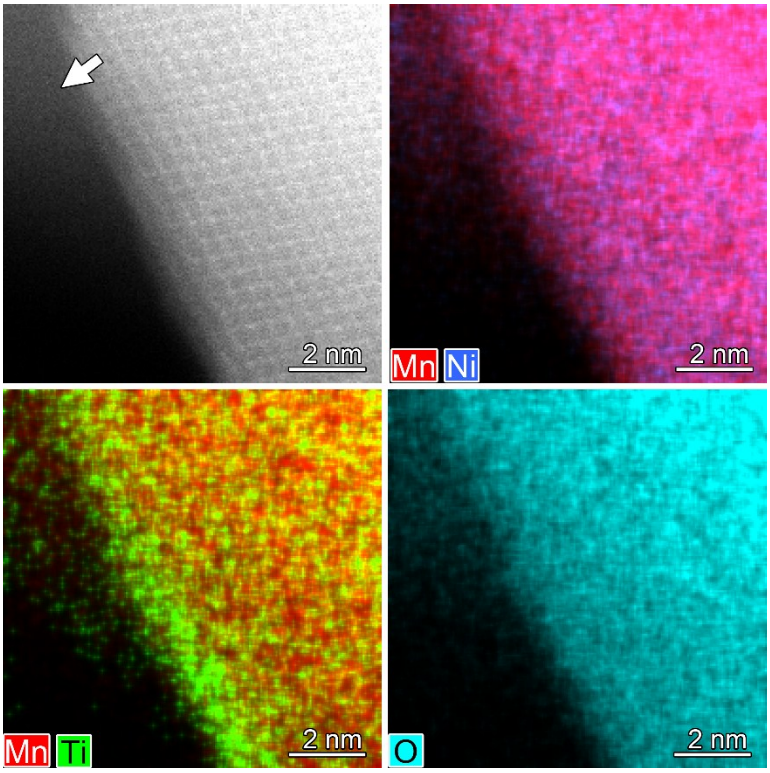

Figure S13. Hıgh resolution HAADF-STEM image, mixed (Mn, Ni), (Mn, Ti) and O STEM-EDX maps of surface modified LNMO annealed at 800°C. Ti layer does not exceed the edges of the crystal, therefore it has spinel structure. The area marked by white arrow corresponds to another crystal.

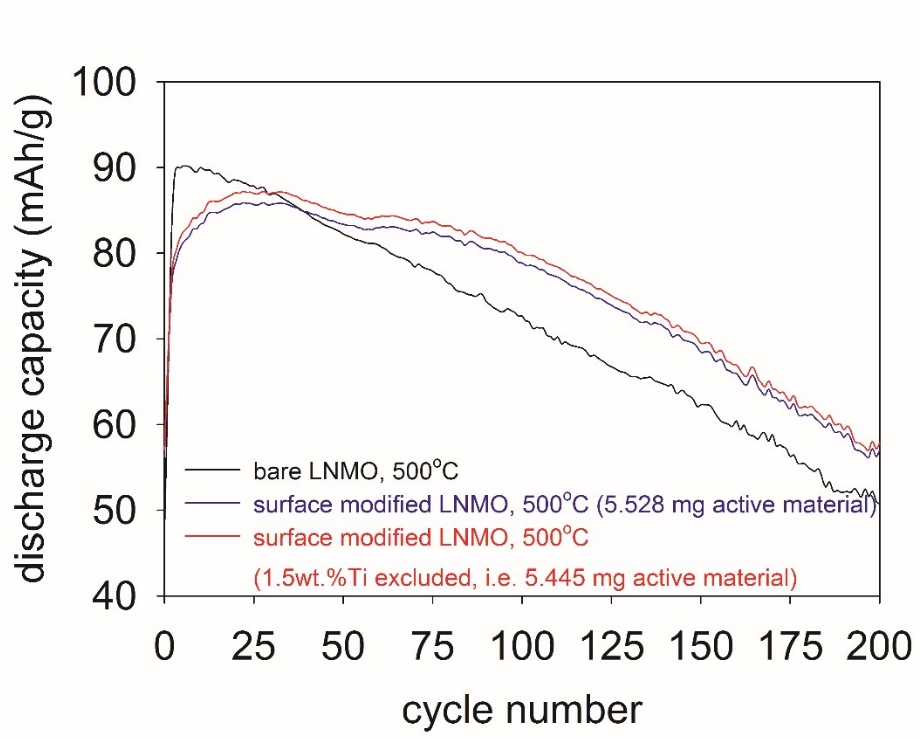

Figure S14. Effect of 1.5wt.% Ti exclusion from the total active material mass on discharge capacity (mAh/g) of surface modified, 500°C annealed LNMO. Since the effect of 1.5wt.% Ti exclusion on discharge capacity (mAh/g) is very small, the Ti was included in the total active material mass for all samples.

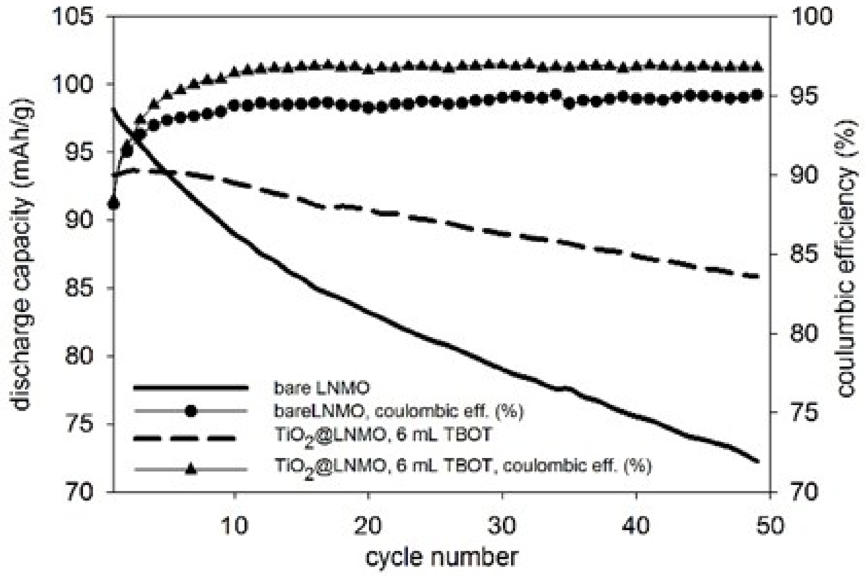

Figure S15. Comparison of cycle life and Coulombic efficiency of the bare LNMO sample without any anneals (as received from Sigma Aldrich) to a surface modified LNMO with 700°C anneal (synthesis made using 3 g LNMO, 6 mL TBOT and 0.5 mL NH_3). Measurements were made using a EC/DEC electrolyte, at 0.1 C, in a potential window from 3.4 to 5 V, for 50 cycles
